# Working Mode and Physical Activity as Factors Determining Stress and Sleep Quality during COVID-19 Pandemic Lockdown in Poland

**DOI:** 10.3390/life12010028

**Published:** 2021-12-24

**Authors:** Anna Lipert, Kamila Musiał, Paweł Rasmus

**Affiliations:** 1 Department of Sports Medicine, Medical University of Lodz, 92-213 Lodz, Poland; kamila.musial@umed.lodz.pl; 2 Department of Medical Psychology, Medical University of Lodz, 90-131 Lodz, Poland; pawel.rasmus@umed.lodz.pl

**Keywords:** working mode, exercise, quality of sleep, health, COVID-19, stress

## Abstract

The coronavirus pandemic and related government restrictions have a significant impact on peoples’ everyday functioning and working, which influences their physical and mental health. The aim of the study was to examine the associations between stress and sleep quality of people of different working modes: working in the workplace (WP), working remotely (RW), and nonworking (NW) in relation to their physical activity (PA) during COVID-19 pandemic lockdown in Poland. It was an online survey performed during governmental lockdown in April 2020. The data were collected form 1959 adults using International Physical Activity Questionnaire—Short Form (IPAQ-SF), Pittsburgh Sleep Quality Index (PSQI), and Perceived Stress Scale (PSS). The conducted analysis included t-Student test, analysis of variance (ANOVA), and mediation analysis (MANOVA). A moderate level of stress was reported in 57% of participants, and 34% of them reported a high stress level. Poor sleep quality was reported in 64% of participants. Total PA performed daily was, on average, 184.8 ± 170.5 min/day for WP, 120.6 ± 124.4 min/day for RW, and 124.6 ± 114.7 min/day for NW (*p* < 0.001). There was a relationship observed between the stress and sleep quality vs. PA habit and working mode, with *p* < 0.05. Being physically active can be beneficial to perceive less stress and sleep disturbances influencing sleep quality, especially in remotely or nonworking people. Planning future pandemic restrictions, the policymakers should be aware of the appropriate guidelines of work planning and PA recommendations for people of different working modes.

## 1. Introduction

In 2019, the global coronavirus pandemic started, which required the introduction of many government restrictions that significantly influenced everyday life of people. There have also been significant changes to the way people work, which has largely been transferred to their homes [1]. The home has become not only a place of silence and relaxation, but, above all, a place of work, especially for white-collar professionals from sectors including government, consulting, academia, accountancy, business, and executive management, etc. [2]. The change of the work organization from those in the workplace to the remote ones is linked with many life and work challenges [3], but, during the pandemic time, a key factor is to avoid the spread of the disease [4]. Unfortunately, if employees were not able to perform their official duties remotely due to the specificity of their work, e.g., blue-collar workers or service workers, it very often resulted in the loss of their jobs [5]. Opportunity of work improves quality of life, mental health, and is a vehicle for improving social inclusion and community tenure [6]. Therefore, a sudden job loss or long-term sick leave are stressors that affect mental health in both men and women [7]. Job insecurity due to restrictions during the coronavirus pandemic has triggered the development of many diseases around European countries, especially those related to mental health, such as post-traumatic disorders, high stress level, depression, or anxiety [8,9]. Those mental disorders, especially stress, are included into risk factors for sleep deprivation and poor quality of sleep [10]. Good quality of sleep and adequate sleep duration necessary for good health of adults 18–60 years of age means sleeping for 7 to 9 h in a 24-h period [11]. For young adults and people with illnesses, more appropriate is sleeping even more than 9 h per night [11]. During the COVID-19 pandemic, a high widespread presence of sleep disturbances was noticed, affecting 40% of the general population and those working in the health care sector [9,12].

The World Health Organization (WHO) has presented a series of recommendations for physical activity to reduce the health consequences of the COVID-19 pandemic [9]. It is evidenced that physical activity (PA) is crucial to maintain general health and so-called well-being by lowering levels of stress, anxiety, and depressed mood [13]. Meta-analytic evidence demonstrates that PA, regardless of the time during the day when it is performed or its intensity, helps to improve sleep quality [14]. Even 10 minutes of moderate intensity walking can improve mood [15] and 10 to 20-min PA bouts lead to cognition improvement [14]. Therefore, even during obligatory social isolation and restrictions of maintaining physical distance, when the previous places for physical activities were no longer accessible, people were encouraged to perform the most simple activities possible to be performed at home. If staying at home was obligatory, simple staircases could serve as a place to perform the simplest physical activity of low intensity. Depending on how fast you go up the stairs, this is a good cardiovascular exercise [16]. The number of publications examining the influence of COVID-19 pandemic governmental restrictions on health, both mental and physical, is still increasing. Studies can be found reporting the implications on emotional and social functioning [17] or the increase in mental health disorders and suicidal incidents [18]. Moreover, some studies can be found presenting the relationship between anxiety, self-efficacy, and stress with the quality of sleep and social support among the population of medical workers or desk workers [19,20,21]. Studies show that the risk factors of mental health disorders during the pandemic are sex, age, economic status, employment status (student or worker), and relationship status [22,23]. However, to our knowledge, there are no studies analyzing the relationship between physical activity, stress, and quality of sleep of the general population during pandemic lockdown and its relation to working mode. Therefore, the aim of the current study was to evaluate the associations between stress and quality of sleep of people of different working modes in relation to their physical activity during the COVID-19 pandemic in Poland. We hypothesize that the working mode performed during pandemic lockdown, along with the physical activity habit, may be of importance in the perceived stress and the obtained quality of sleep obtained.

## 2. Materials and Methods

### 2.1. Study Design and Data Collection

The study data were obtained from the 1959 adult respondents at the age of 18 years and over. An anonymous online survey was designed using Google Forms and the link was distributed by social media, such as Facebook or Twitter. Moreover, the survey was distributed through communicational tools, such as Messenger, WhatsApp, or email. Furthermore, the most popular influencers were invited to the study to encourage participation in the survey. After activating the link to the survey questions, there was a content informing that the study was fully voluntary, anonymous, and unpaid. Due to the anonymity of the questionnaire and the fact that its completion was equivalent to agreeing to participate in the study, the relevant University Human Ethics Committee decided to exempt it from its approval. The research was performed between 1 and 14 April 2020 during governmental lockdown. The study meets the relevant standards of the journal.

The presented study is a further analysis from data previously published [24], which were focused on the general population without taking into account the working mode performed during the pandemic lockdown.

The governmental lockdown restrictions in the country during the time of the study required keeping a minimum 2 m personal distance, and there were travel restrictions limiting the amount of people in public transport. Traveling was allowed only for essential workers or services, such as medical or healthcare. People were not allowed to leave their place of residence, except in necessary situations, such as getting to work or necessary shopping. Children and adolescents were not allowed to leave the house without the supervision of an adult. Any form of public gathering was also prohibited. There were designated special hours between 10 and 12 a.m. for seniors in the shops, so any person below 65 years was not allowed to enter. All the services, such as restaurants and bars, hotels, parks, hairdressers, and beauty studios, were closed. There was only an online learning allowed at schools and universities.

### 2.2. Measurement Instruments

To obtain the socio-demographic data and relevant information most influencing the physical activity, quality of sleep, and perceived stress, a self-made questionnaire was designed.

International Physical Activity Questionnaire—Short Form (IPAQ-SF) was used to collect information about the physical activity performed during the last typical week [25,26]. The questionnaire concerns physical activity of three different intensities: low physical activity in the form of walking (WPA = 3.3 METs), moderate physical activity (MPA = 4.0 METs), and vigorous physical activity (VPA = 8.0 metabolic equivalent (METs)) and across a set of domains, such as work-related activities, transport-related activities, leisure time, and domestic and gardening (yard) activities. The final results were calculated according to the IPAQ scoring protocol guideline [26,27] and presented as the total minutes of PA per day.

Pittsburgh Sleep Quality Index (PSQI) was used to collect the data related to the quality and patterns of sleep over the last month. The questionnaire is designed to measure seven components: subjective sleep quality, sleep latency and its duration, habitual efficiency of sleep, sleep disturbances, sleeping medication usage, and daytime dysfunction, and is able to differentiate “poor” from “good” quality of sleep. The results are presented in points and, if the final score is “5” or greater, it is indicated with poor sleep quality. Studies conducted on various populations have shown the strong reliability and validity of PSQI, which suggests that this tool fulfils its intended utility [28].

Perceived Stress Scale (PSS) [29,30] was used to collect the data about the perceived stress during the last month. The PSS was originally developed in 1983 and, since then, it has been a validated stress assessment tool. The results are presented in points, which can range from 0 to 40. Higher scores indicate higher perceived stress. The ranges are as follows: 0–13 points means a low level of stress (LLS); 14–26 points means a moderate level of stress (MLS), and 27–40 points means a high level stress (HLS).

All of the measurement instruments were translated into Polish and pretested to check if the questions were well understood in the mother tongue. Moreover, after the questions were posted on Google Forms, it was checked whether the link was working properly and that it was possible to provide full answers. Only then was the link distributed through the previously described channels.

### 2.3. Statistical Analysis

All the statistics were performed using Statistical version 13.1 software (StatSoft). When the Shapiro–Wilk test revealed that the variables had a normal distribution and there was a homogeneity of variances, the differences were analyzed using t-Student test for two quantitative variables or ANOVA for more than two variables. The moderation analysis of variance (MANOVA) was also performed to observe the relationship between the stress and sleep results vs. the type of working mode and the habit of undertaking PA. Mann–Whitney test was used if the variables were not normally distributed. The results were presented as mean scores and standard deviations. All the differences at the level of *p* < 0.05 were accepted as statistically significant.

## 3. Results

### 3.1. Characteristics of the Participants

**Figure 1:**
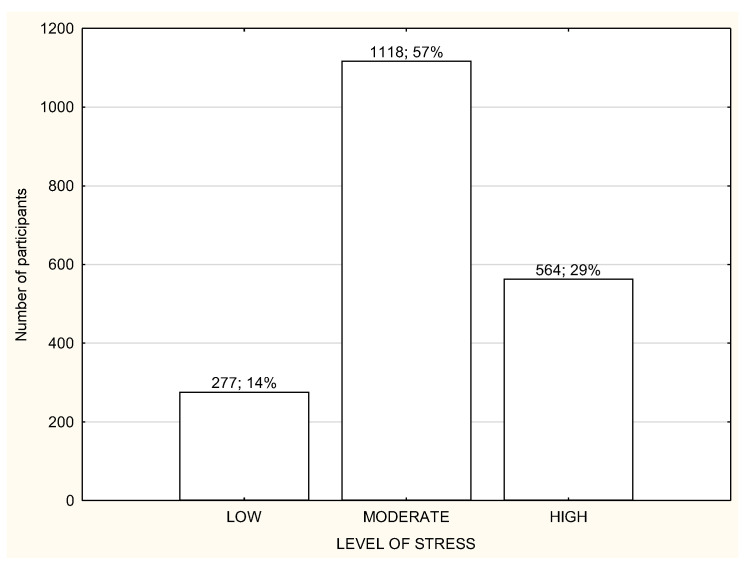
Level of stress among the study participants (*n* = 1959).

**Figure 2:**
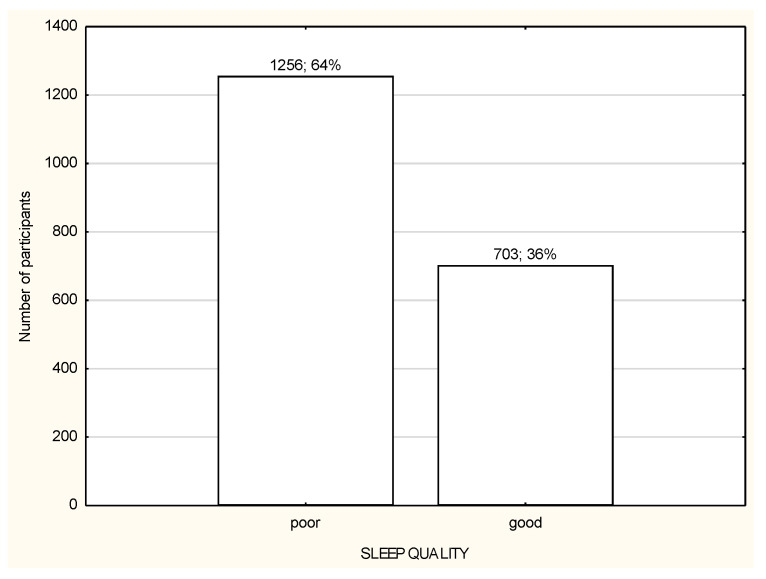
Quality of sleep among the study participants (*n* = 1959).

The socio-demographic characteristics of the study participants are presented in Table 1. The study participants were divided into three groups according to the working mode during lockdown: (1) working in the workplace (WP); (2) working remotely (RW); (3) not working or unemployed (NW). A large portion of the study participants from all three groups declared living in the big city and performing office work (Table 1). Most of the respondents were subject to general governmental restrictions (Table 1). Most of the study participants in all three groups reported a moderate or high level of stress and were usually characterized by poor quality of sleep (Figure 1 and Figure 2). Over a half of the participants in every group declared being physically active during lockdown (Table 1). The total habitual physical activity performed during the day was, on average, 184.8 ± 170.5 min/day for WP in comparison to 120.6 ± 124.4 min/day for RW and 124.6 ± 114.7 min/day for NW and the difference was statistically significant (*p* < 0.001) (Table 2). In the whole study group, the less active seemed to be RW participants (Table 2). People who declared being physically active had significantly more total daily time of PA in comparison to inactive people, especially RW and NW (Table 2). There were no differences in total daily time of PA and walking time PA between active and inactive WP; there were significant differences in moderate and vigorous PA time, with *p* < 0.001 (Table 2).

### 3.2. Level of Stress in Relation to Working Mode during Lockdown

**Figure 3:**
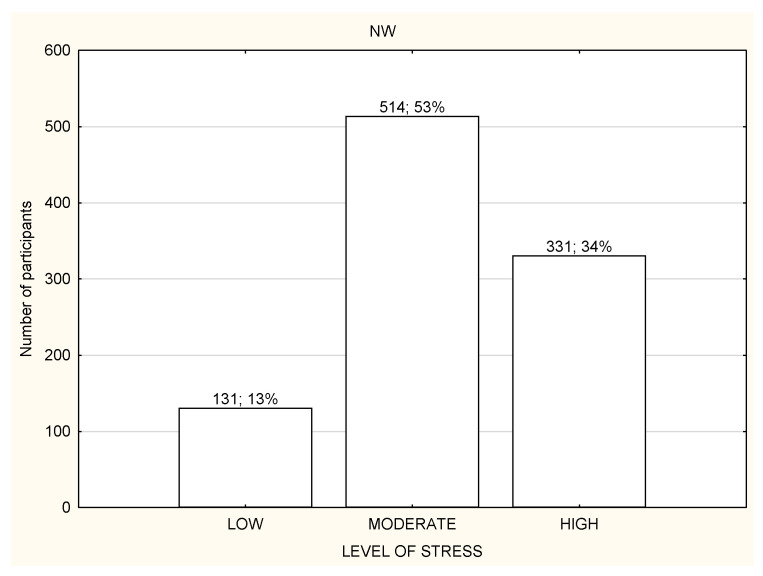
Level of stress among the nonworking participants (*n* = 1959).

**Figure 4:**
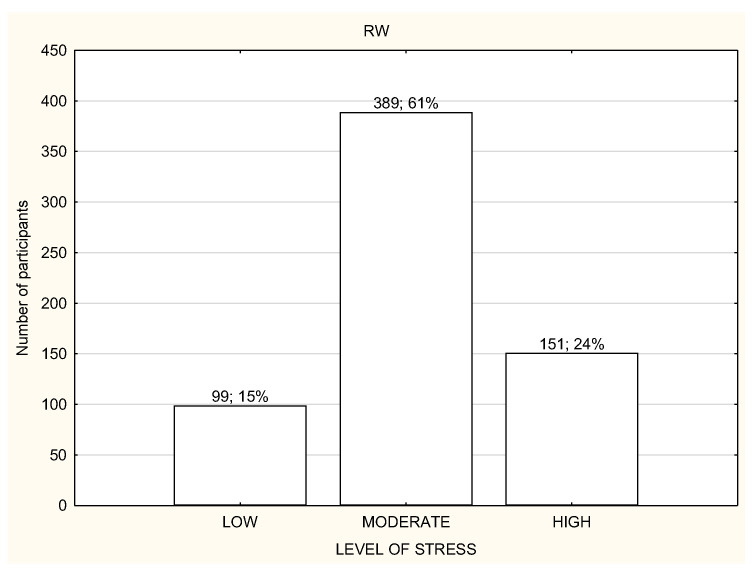
Level of stress among the participants working remotely (*n* = 1959).

**Figure 5:**
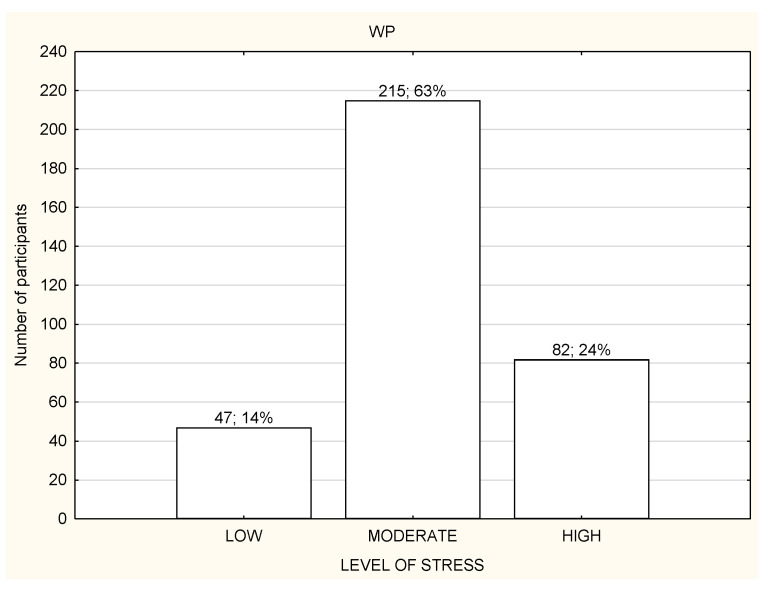
Level of stress among the participants working in the workplace (*n* = 1959).

Most of the participants had a moderate or high level of stress regardless of the working mode (Figure 3, Figure 4 and Figure 5). However, NW participants were noticed to have a significantly higher level of stress in the PSS results in comparison to RW and WP, with *p* < 0.01 (Table 3). NW was characterized with the worst results in all the components of stress assessed by PSS, and the differences were statistically significant (Table 3). The results of RW and WP were similar, except two components: being upset because of something that happened unexpectedly and feeling unable to control the important things in life, but the differences were not statistically significant (Table 3).

### 3.3. Quality of Sleep in Relation to Working Mode during Lockdown

**Figure 6:**
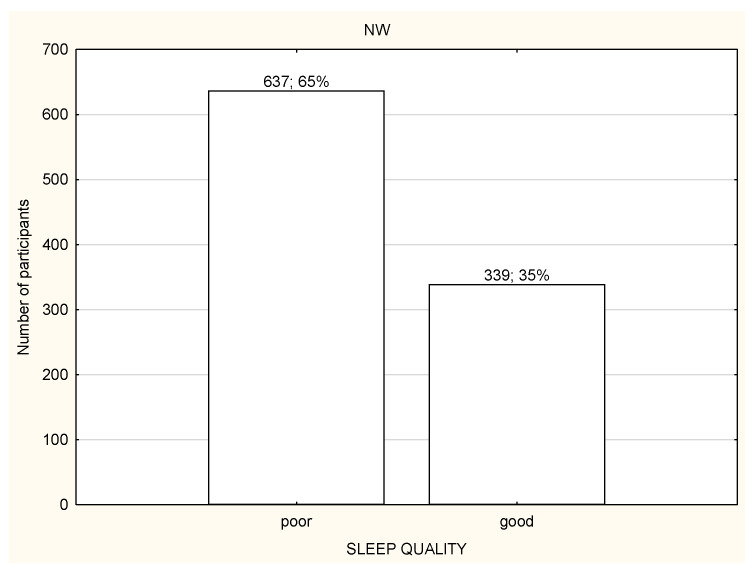
Quality of sleep among the nonworking participants (*n* = 1959).

**Figure 7:**
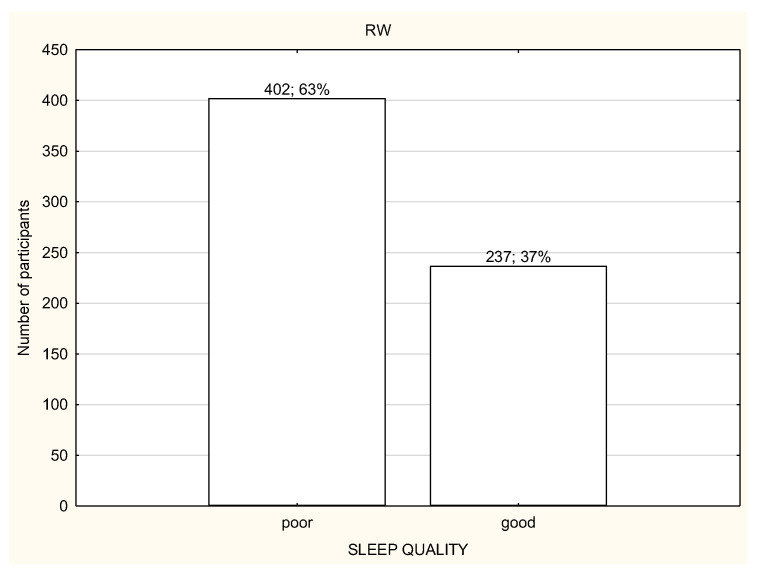
Quality of sleep among the participants working remotely (*n* = 1959).

**Figure 8:**
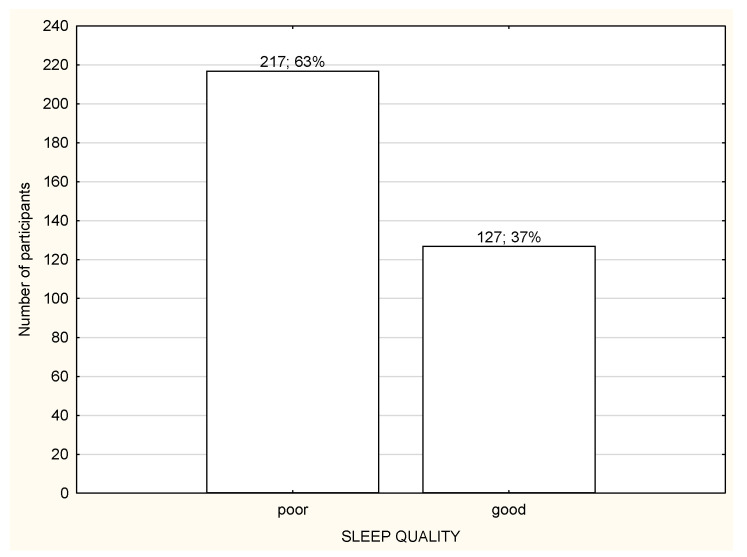
Quality of sleep among the participants working in the workplace (*n* = 1959).

Over a half of the study participants reported poor quality of sleep (Figure 6, Figure 7 and Figure 8) and there were no differences in the quality of sleep in the PSQI results between the NW, RW, and WP (Table 4). Moreover, NW was characterized with significantly worse sleep latency (*p* < 0.05) in comparison to RW and WP (Table 4). WR was characterized with the best sleep latency and less sleep disturbances in comparison to NW and WP, but the differences were not statistically significant. However, RW was observed with the highest use of sleeping medications in comparison to NW and WP (Table 4).

### 3.4. Perceived Stress and Sleep Quality Depended on the Working Mode and Undertaken PA during Lockdown

**Figure 9:**
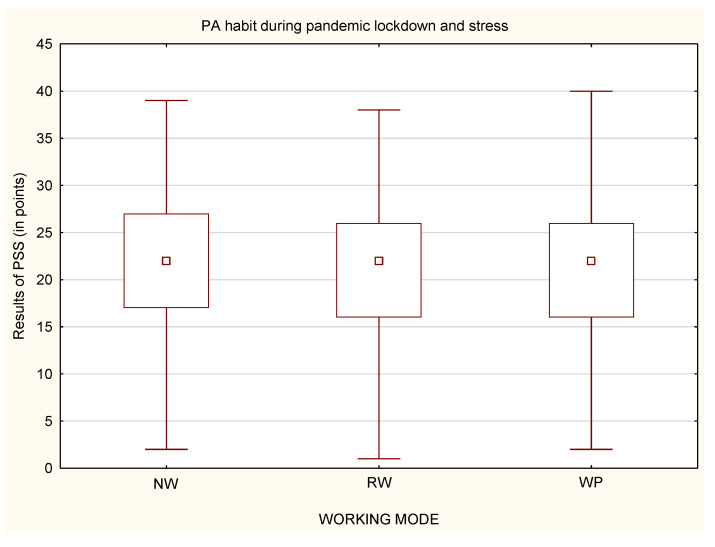
Differences in the level of stress in relation to working mode among physically active participants (*n* = 1959).

**Figure 10:**
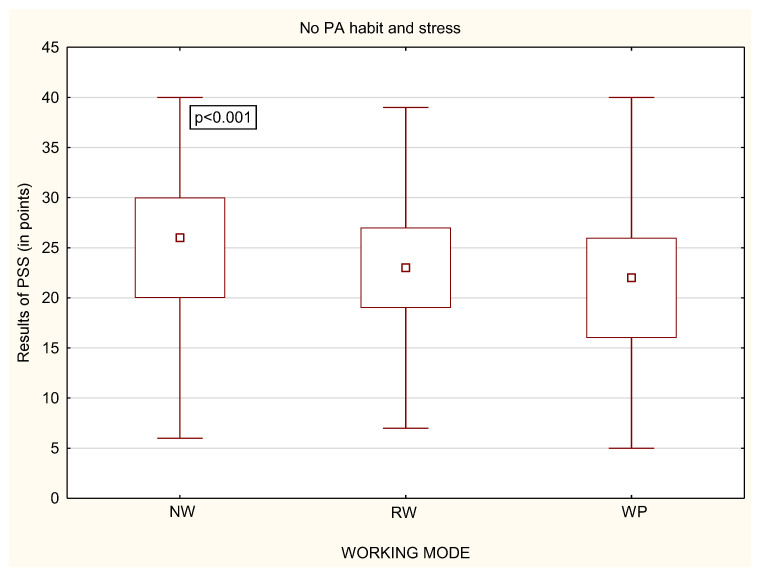
Differences in the level of stress in relation to the working mode among physically inactive participants (*n* = 1959).

While analyzing whether a person declared to undertake physical activity or not, it was noticed that, among physically active people, there were no statistically significant differences in the perceived stress, regardless of the working mode (Figure 9). Statistically significant differences were only in the group of physically inactive people (Figure 10). Physically inactive NW were characterized by the highest level of perceived stress in comparison to physically inactive RW and WP with *p* < 0.001 (Figure 10). The MANOVA analysis, which takes into account multiple variables, confirmed the relationship between stress, PA habit and all of the forms of working mode, with *p* < 0.001.

**Figure 11:**
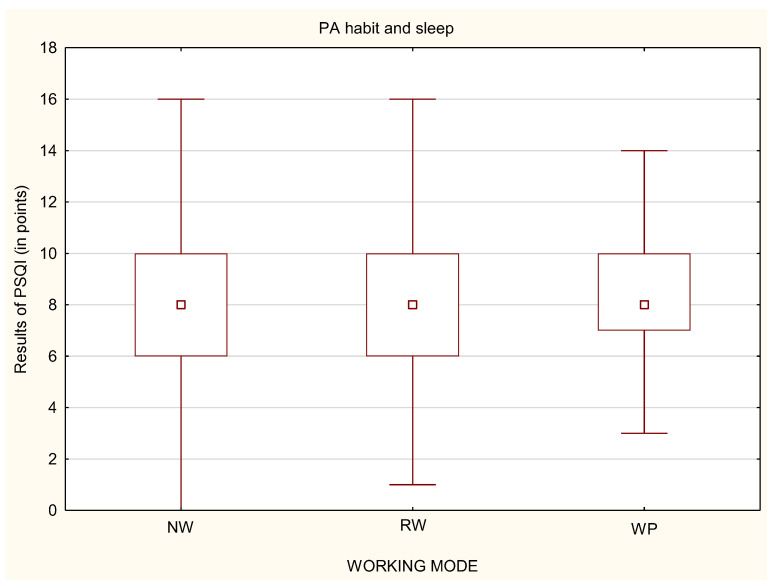
Differences in the quality of sleep in relation to the working mode among physically active participants (*n* = 1959).

**Figure 12:**
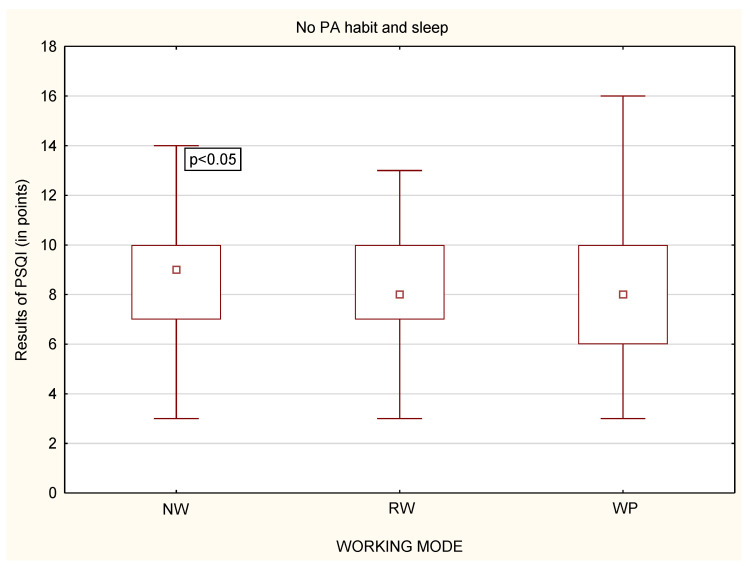
Differences in the quality of sleep in relation to the working mode among physically inactive participants (*n* = 1959).

Similarly to stress level, while analyzing whether a person declared to undertake physical activity or not, it was noticed that, among physically active people, there were no statistically significant differences in the quality of sleep, regardless of the working mode (Figure 11). Statistically significant differences were only in the group of physically inactive people (Figure 12). Physically inactive NW were characterized by poorer quality of sleep in the PSQI results in comparison to physically inactive RW and WP with *p* < 0.05 (Figure 12). The MANOVA analysis confirmed the relationship between stress, PA habit, and remote work (*p* < 0.05) or nonworking people (*p* < 0.01) (Table 5).

## 4. Discussion

The COVID-19 pandemic caused a lot of changes in the working mode of many people. Some of them lost their job or were forced to change it, but a lot of people changed the organization of work from being in the workplace to remote work at home. Different working modes, the coexisting pandemic situation, and obligatory isolation were not neutral for physical and mental health and can be a reason for serious mental disorders [31], and even suicidal thoughts [18].

The aim of the study was to examine the associations between stress and quality of sleep in relation to physical activity performed by people of working modes during COVID-19 pandemic lockdown in Poland.

In the present study, a high level of stress was significantly noticed more often in unemployed people in comparison to employed people. A systematic review also confirms that, during the pandemic, a depression risk factor is observed more often among unemployed people [32]. People who are employed were usually characterized with moderate stress level. The reason for that difference may be due to the fact that the unemployed people spent more time on reading newspapers, watching TV, or listening to the radio, so the media channels that were delivering huge amounts of information about the current world epidemiological situation could increase fear, anxiety, and, thus, the level of stress. This, in conjunction with the governmental restrictions prohibiting leaving the place of residence, limited social contact with people and general disturbance of everyday life could influence both stress level and sleep quality, especially those unemployed. Moreover, unemployed people had to be financially dependent on other people (relatives; friends) and, therefore, they may not receive adequate medical care in the event of an infection of COVID-19. All described factors are thought to negatively affect mental health [33]. Employed people could feel more safety during possible COVID-19 infection due to the possession of health insurance; additionally, people working in the workplace had more social contact with other workmates, a very important aspect in decreasing stress. People who had to change their working mode from work in the workplace to remote work reported perceiving more stress; they were characterized with “work-life balance” (WLB) disorder and a decrease in work satisfaction, [34]. To reduce the stress level, an increase in alcohol consumption was observed, together with other addictive substances, which also had a negative impact on physical and mental health [5,35].

The difficulty in achieving “work-life balance” by remotely working people was also observed in the previous study [34] before the pandemic [6]. Therefore, it is recommended that people working remotely should have some separate rules organizing their work and life to achieve WLB [36].

The pandemic situation was also responsible for sleep disturbances, such as falling asleep unintentionally, difficulties falling/staying asleep, and later bedtime, often using sleep medication [35]. Our study showed that sleep quality was poor among both employed and unemployed people. Indeed, there were no significant differences between unemployed–employed people and remotely working–working in the workplace people. Other study confirmed that, despite the fact that people sleep longer and spend more time in bed during pandemic lockdown than before the COVID-19 pandemic, their sleep quality decreases [37,38].

Governmental restrictions modifying the everyday life of people limited access to the routine classes and sport facilities, decreasing the general PA level. What was also visible in the present study was people who were unemployed but physically active had better sleep quality than those who were unemployed and physically inactive.

Employed people working in the workplace spent statistically significantly more time on physical activity in comparison to people with remote work and unemployed people. It was also connected with the fact that people working in the workplace had to spend some time on travel to the job destination. If the work was close enough to home that it allows you to travel along the way, it gave them the additional option of undertaking PA.

The results of the present study showed that lower stress level was observed among unemployed people who declared to be physically active in comparison to those who were physically inactive. On the other side, the research shows that that the high stress level could be a cause of low PA because of the lack of interest and motivation [39]. There are a lot of advantages of regular PA and its link with mental health improvement during isolation [40,41]; during the pandemic, the most important seems to be to decrease the risk or mellow the course of infection and immunology system improvement [42], rather than maintaining PA in the community.

The employers should provide mental support for their employees [20], with special attention paid to the psychological help, especially among the healthcare providers. It was shown that healthcare professionals who were closely working with COVID-19-positive people were noticed to have an increased stress level and decreased sleep quality [14,19]. Moreover, they reported anxieties and depression [43].

The main strength of the study is its large sample. Next, the study was performed during a special time of pandemic lockdown, which made it possible to analyze people’s behavior and well-being at that particular time. Further, the data were collected using internationally recognized and validated tools. Moreover, the online form of the study made it possible to provide access to many people during pandemic lockdown.

However, some limitations of the study should be emphasized. Firstly, it was a survey study using questionnaires, so the results are more subjective and may be underestimated or overestimated by the answers of the respondents. It is usually recommended that a study in the form of interview with the questionnaire is conducted in the presence of an investigator who can sometimes help clarify the question. Unfortunately, during the pandemic lockdown, the access and direct contact with people was very reduced. Secondly, in the study group, there is a fairly large advantage of women over men. Probably, this is due to the fact that, in surveys, especially those online, it is noticed that women participate much more often rather than men.

To summarize, the pandemic lockdown caused people to feel a lot of stress and significantly worsened their quality of sleep, regardless of the working mode. However, being unemployed was associated with a greater risk of experiencing a lot of stress and sleep disturbance. It was also noted that physical activity, even at home during a lockdown, can help reduce stress and, consequently, improve the quality of sleep, which was especially visible among the unemployed. Maintaining the possibility of active work in any form, remotely or in the workplace, and undertaking physical activity translate into a reduction in stress and improve the quality of sleep. The presented conclusions should be borne in mind when there is another need to introduce such large restrictions in the everyday functioning of people.

### Practical Implications

The study could be a recommendation for employers, informing what type of work should be implemented for their employee during pandemic time to maintain mental health, which is linked with quality of work. The study results should be valuable for hospital authorities to reduce the mental health burden of healthcare workers associated with COVID-19.

## 5. Conclusions

During pandemic lockdown, people were overstressed and had a poor quality of sleep. However, regardless of the working mode, people who had the opportunity to stay professionally active perceived less stress than nonworking people. However, being physically active can be beneficial to perceiving less stress and sleep disturbances influencing sleep quality, especially among remotely working and nonworking people. Planning future pandemic restrictions, the policymakers should be aware of designing the appropriate guidelines of work planning and physical activity recommendations regarding the different working modes.

## Figures and Tables

**Table 1:** The main socio-demographic variables characterizing the study group (*n* = 1959).

SOCIO-DEMOGRAPHIC VARIABLES	WP (N = 344)	RW (N = 639)	NW (N = 976)
	*n* (%)		
Sex			
Female	270 (78.5)	533 (83.4)	878 (90.0)
Male	74 (21.5)	106 (16.6)	98 (10.0)
Place of living			
City >100,000 citizens	163 (47.4)	380 (59.5)	456 (46.7)
City 20–100,000 citizens	80 (23.2)	101 (15.8)	198 (20.3)
Town <20,000 citizens	41 (12.0)	42 (6.6)	97 (10.0)
Village	60 (17.4)	116 (18.1)	225 (23.0)
Type of work			
Office work	210 (61.0)	555 (86.8)	384 (39.3)
Physical work	132 (38.4)	31 (4.8)	282 (29.0)
Not applicable	2 (0.6)	53 (8.3)	310 (31.8)
Form of restriction during pandemic			
Governmental restrictions	210 (61.0)	529 (82.8)	803 (82.3)
Quarantine	0 (0)	7 (1.1)	16 (1.6)
No restrictions because of the type of occupation (medical staff, etc.)	134 (39.0)	103 (16.1)	157 (16.1)
Undertaking physical activity during pandemic			
YES	209 (60.8)	462 (72.3)	700 (71.7)
NO	135 (39.2)	177 (27.7)	276 (28.3)

**Table 2:** Amount of time of physical activity performed by the study participants (*n* = 1956) who declared performing physical activity and being inactive.

	WP (N = 344)	RW (N = 639)	NW (N = 976)
	Mean (±SD)		
Total PA (min/day)	184.8 ± 170.5 ***^,###^	120.6 ± 124.4	124.6 ± 114.7
Walking PA (min/day)	39.9 ± 35.6 *^,#^	27.9 ± 32.0	30.0 ± 32.4
Moderate PA (min/day)	7.5 ± 9.7	7.6 ± 9.4	8.3 ± 9.8
Vigorous PA (min/day)	9.4 ± 11.8	9.4 ± 12.3	9.7 ± 12.1
PEOPLE PHYSICALLY ACTIVE			
Total PA (min/day)	183.4 ± 152.4 ***^,###^	129.1 ± 114.3	130.4 ± 103.6
Walking PA (min/day)	39.5 ± 35.6 *^,#^	29.4 ± 31.9	30.8 ± 32.4
Moderate PA (min/day)	9.9 ± 9.9	9.8 ± 9.6	10.6 ± 10.1
Vigorous PA (min/day)	13.6 ± 12.5	12.3 ± 13.1	12.8 ± 12.5
PEOPLE PHYSICALLY INACTIVE			
Total PA (min/day)	186.8 ± 195.8 ***^,###^	98.4 ± 145.7 ^^^^^	109.9 ± 138.1 ^^^^^
Walking PA (min/day)	40.6 ± 35.9 *^,#^	24.1 ± 31.9	27.9 ± 32.2
Moderate PA (min/day)	3.9 ± 8.5 ^^^^^	2.3 ± 6.3 ^^^^^	2.9 ± 6.1 ^^^^^
Vigorous PA (min/day)	2.5 ± 6.1 ^^^^^	1.9 ± 5.1 ^^^^^	2.2 ± 6.4 ^^^^^

* *p* < 0.05; *** *p* < 0.001 IN THE WORK PLACE vs. REMOTE WORK. ^#^ *p* < 0.05; ^###^ *p* < 0.001 IN THE WORK PLACE vs. NOT WORKING/UNEMPLOYED. ^^^ *p* < 0.001 ACTIVE vs. INACTIVE.

**Table 3:** Level of stress (mean ± SD) estimated by the Perceived Stress Scale (PSS).

	WP (N = 344)	RW (N = 639)	NW (N = 976)
	Mean (±SD)		
Overall level of stress (Perceived Stress Scale; PSS) (AU)	21.5 ± 7.1	21.5 ± 7.2	22.6 ± 7.5 **^,##^
1. Been upset because of something that happened unexpectedly (AU)	2.3 ± 1.0	2.1 ± 1.0	2.2 ± 1.0 *
2. Felt that you were unable to control the important things in your life (AU)	1.9 ± 1.1	2.1 ± 1.1	2.2 ± 1.2 *^,###^
3. Felt nervous and “stressed” (AU)	2.4 ± 1.0	2.4 ± 1.0	2.5 ± 1.0
4. Felt confident about your ability to handle your personal problems (AU)	2.0 ± 1.0	2.0 ± 1.0	2.2 ± 1.0 ***^,##^
5. Felt that things were going your way (AU)	2.3 ± 0.9	2.3 ± 0.9	2.5 ± 0.9 ***^,##^
6. Found that you could not cope with all the things that you had to do (AU)	1.8 ± 1.0	1.8 ± 1.1	2.0 ± 1.1 **^,##^
7. Been able to control irritations in your life (AU)	1.9 ± 0.9	1.9 ± 1.0	2.0 ± 1.0 *
8. Felt that you were on top of things (AU)	2.8 ± 0.9	2.8 ± 1.0	2.9 ± 1.0 *
9. Been angered because of things that were outside of your control (AU)	2.1 ± 1.1	2.2 ± 1.1	2.2 ± 1.1 ^#^
10. Felt difficulties were piling up so high that you could not overcome them (AU)	1.7 ± 1.2	1.7 ± 1.2	1.9 ± 1.2 ***^,##^

* *p* < 0.05; ** *p* < 0.01; *** *p* < 0.001 WR vs. NW. ^#^ *p* < 0.05; ^##^ *p* < 0.01; ^###^ *p* < 0.001 WP vs. NW. AU: arbitrary units.

**Table 4:** Subjective sleep quality (mean ± SD) estimated by the Pittsburgh Sleep Quality Index (PSQI).

	WP (*n* = 344)	RW (*n* = 639)	NW (*n* = 976)
Quality of sleep (Global PSQI Score) (AU)	8.21 ± 2.78	8.19 ± 4.30	8.26 ± 2.64
Component 1: subjective sleep quality (AU)	1.33 ± 0.85	1.24 ± 0.89	1.28 ± 0.86
Component 2: sleep latency (AU)	1.34 ± 1.03	1.27 ± 0.99	1.38 ± 1.03 *
Component 3: sleep duration (AU)	2.63 ± 0.95	2.66 ± 0.89	2.59 ± 0.99
Component 4: habitual sleep efficiency (AU)	0.80 ± 1.20	1.11 ± 4.62	0.99 ± 3.21
Component 5: sleep disturbances (AU)	0.95 ± 0.21	0.94 ± 0.23	0.96 ± 0.19
Component 6: use of sleeping medications (AU)	0.24 ± 0.71	0.27 ± 0.73	0.19 ± 0.62 *
Component 7: daytime dysfunction over the last month (AU)	1.03 ± 0.88	1.10 ± 0.86	1.12 ± 0.87

*: Significant difference between RW and NW. AU: arbitrary units.

**Table 5:** ANOVA/MANOVA analysis results.

	Type III Sum of Squares	df	Mean Square	F	*p* Value
STRESS					
(Intercept)	705,269.9	1	705,269.9	13,242.12	0.000000
Work	989.5	2	494.7	9.29	0.000097
PA	639.1	1	639.1	12.00	0.000544
Work × PA	389.4	2	194.7	3.66	0.026026
Error	104,016.0	1953	53.3		
SLEEP					
(Intercept)	99,053.36	1	99,053.36	9196.543	0.000000
Work	13.58	2	6.79	0.630	0.532591
PA	49.88	1	49.88	4.632	0.031512
Work × PA	41.36	2	20.68	1.920	0.146880
Error	21,002.90	1950	10.77		

## Data Availability

The datasets used and/or analyzed during the current study are available from the corresponding author on reasonable request.
